# Potentiated inhibition of *Trichoderma virens* and other environmental fungi by new biocide combinations

**DOI:** 10.1007/s00253-021-11211-3

**Published:** 2021-03-18

**Authors:** Cindy Vallières, Cameron Alexander, Simon V. Avery

**Affiliations:** 1 School of Life Sciences, University of Nottingham, University Park, Nottingham, NG7 2RD UK; 2 School of Pharmacy, University of Nottingham, University Park, Nottingham, NG7 2RD UK

**Keywords:** Fungicide, Biodeterioration, Biodegradation, *Aspergillus brasiliensis*, *Penicillium funiculosum*, *Chaetomium globosum*, *Aureobasidium pullulans*

## Abstract

**Abstract:**

Fungi cause diverse, serious socio-economic problems, including biodeterioration of valuable products and materials that spawns a biocides industry worth ~$11 billion globally. To help combat environmental fungi that commonly colonise material products, this study tested the hypothesis that combination of an approved fungicide with diverse agents approved by the FDA (Food and Drug Administration) could reveal potent combinatorial activities with promise for fungicidal applications. The strategy to use approved compounds lowers potential development risks for any effective combinations. A high-throughput assay of 1280 FDA-approved compounds was conducted to find those that potentiate the effect of iodopropynyl-butyl-carbamate (IPBC) on the growth of *Trichoderma virens*; IPBC is one of the two most widely used Biocidal Products Regulations–approved fungicides. From this library, 34 compounds in combination with IPBC strongly inhibited fungal growth. Low-cost compounds that gave the most effective growth inhibition were tested against other environmental fungi that are standard biomarkers for resistance of synthetic materials to fungal colonisation. Trifluoperazine (TFZ) in combination with IPBC enhanced growth inhibition of three of the five test fungi. The antifungal hexetidine (HEX) potentiated IPBC action against two of the test organisms. Testable hypotheses on the mechanisms of these combinatorial actions are discussed. Neither IPBC + TFZ nor IPBC + HEX exhibited a combinatorial effect against mammalian cells. These combinations retained strong fungal growth inhibition properties after incorporation to a polymer matrix (alginate) with potential for fungicide delivery. The study reveals the potential of such approved compounds for novel combinatorial applications in the control of fungal environmental opportunists.

**Key points:**

*• Search with an approved fungicide to find new fungicidal synergies in drug libraries.*

*• New combinations inhibit growth of key environmental fungi on different matrices.*

*• The approach enables a more rapid response to demand for new biocides.*

**Supplementary Information:**

The online version contains supplementary material available at 10.1007/s00253-021-11211-3.

## Introduction

The fungi include major pathogens of humans, plants and livestock, food spoilage organisms, and organisms causing deterioration of synthetic products and materials (Fisher et al. 2020; Meyer et al. 2016). Therefore, they have substantial economic and societal importance. Fungal that colonise products and materials and may cause their deterioration are characterised by high rates of spore dissemination to the surrounding environment, ability to colonise diverse substrates and, depending on the species, the potential for mycotoxin production and pathogenicity (Pitkaranta et al. 2011). Furthermore, approximately 10% of people worldwide suffer allergies to environmental fungi (Chakravarty and Kovar 2013; Robbins et al. 2000).

Biocides are important for preventing biodeterioration of a variety of material products including plastics, paints and coatings, metalworking fluids, textiles, building materials, wood, and cosmetics. All of these are susceptible to fungal colonisation. However, the actions of biocides against fungi are less well characterised than against bacteria, and also compared with antifungal drugs that are used for treating disease. In general, fungi are more resistant to biocides than nonsporulating Gram-positive bacteria, but can be more sensitive than Gram negative bacteria (McDonnell and Russell 1999). Halogen compounds are a key biocide class, followed by metallic compounds and organosulfurs. However, there are growing environmental and regulatory concerns over the uses of metallic or halogenated compounds containing bromine and chlorine, and stringent regulations regarding their use are likely to accentuate the need for alternative biocide treatments (https://echa.europa.eu/legislation). Developing new biocides involves high costs and takes time given the regulatory hurdles, which does not encourage such investment. An alternative route for the industry could be development of new blends of existing biocides. For example, the combination of iodopropynyl-butyl-carbamate (IPBC) and 2-n-octyl-4-isothiazolin-3-ones (OIT) is used in current commercial preservatives, for water-borne dry film interior coatings. IPBC is a water-soluble, halogenated and unsaturated carbamate derivative that exhibits strong fungicidal activity. It is a Biocidal Products Regulations (BPR)–approved fungicide that has been in use for many years as a wood and paint preservative. IPBC has also found applications in the cosmetics industry (Maier et al. 2009; Steinberg 2002) and has been approved by the Food and Drug Administration (FDA) as an adhesive component for use in food packaging and storage. However, several cases of allergic contact dermatitis have been related to IPBC use (Badreshia and Marks Jr 2002; Vanhoutte et al. 2019).

In this study, it was hypothesised that a much more diverse range of agents could have potential use against environmental fungi in combination with IPBC. Such agents were screened for in a FDA-approved compound library. The re-purposing of existing, approved drugs for alternative uses has emerged as a key approach for facilitating therapeutic drug development (Holbrook et al. 2017; Robbins et al. 2015; Roemer and Krysan 2014; Spitzer et al. 2011; Vallieres et al. 2020b). Here, this strategy was adapted for biocide discovery. The study reports new combinations that effectively and selectively target fungi of the ASTM G21 group—the standard used by the industry for determining resistance of synthetic polymeric materials to fungi (www.astm.org/cgi-bin/resolver.cgi?G21)—including after incorporation to a polymer matrix, supporting the potential for applications.

## Methods

### Strains, culture, and maintenance

Fungal strains used in this study were the moulds *Aspergillus brasiliensis* ATCC 9642, *Penicillium funiculosum* ATCC 11797, *Chaetomium globosum* ATCC 6205, *Aureobasidium pullulans* ATCC 15233, and *Trichoderma virens* ATCC 9645 from the ASTM G21 group (www.astm.org/cgi-bin/resolver.cgi?G21). The fungi were inoculated from spore stocks kept at − 80 °C (in potato dextrose broth (PDB; Sigma, St Louis, MO, USA) and 20% glycerol) onto potato dextrose agar (PDA; Oxoid, Basingstoke, UK) and cultured for 7 days at room temperature. Using a sterile cotton bud, spores were transferred from these starter plates to new PDA plates. Starter plates were kept at 4 °C for a maximum of 3 weeks. Fungi on the new plates were grown for 7 days at room temperature before spores were harvested in PDB and filtered through a 40-μm cell strainer for experimental use. The collected spores were checked for purity by microscopy and counted using a hemocytometer. Growth assays (below) were performed in PDB at 30 °C.

### High-throughput screening

Compounds of the Prestwick Chemical Library (PL) (Prestwick Chemical, Illkirch-Graffenstaden, France) were used at a final concentration of 100 μM in the presence or absence of 200 ng ml^−1^ IPBC (Sigma). Screens were performed using *T. virens* spores inoculated from 7-day PDA plates to PDB broth adjusted to a final concentration of 15,000 spores ml^−1^. Aliquots (100 μl) of the spore suspension in PDB, plus chemical supplements as above, were transferred to 96-well plates (Greiner Bio-One, Stonehouse, UK) and cultured for 72 h at 30 °C with OD_600_ measured at 40 h and 72 h in a BioTek EL800 microplate spectrophotometer. OD_600_ was expressed as percentage of growth relative to control growth (i.e., without added IPBC or PL compound). Effect strength ((% growth with PL compound) minus (% growth with PL compound + IPBC)) was calculated for each combination; screen hits were considered those agents showing an effect strength >50.

One thousand compounds from the University of Nottingham Managed Chemical Compound Collection (NMCCC) were also tested in combination with IPBC against the yeast *Saccharomyces cerevisiae* BY4743. *S. cerevisiae* was grown overnight in YPD broth (2% peptone (Oxoid), 1% yeast extract (Oxoid), 2% D-glucose), diluted to OD_600_ ~0.5 and cultured for a further 4 h in fresh medium before being diluted again to OD_600_ ~0.1 as described previously (Moreno-Martinez et al. 2015). Aliquots (150 μl) of the diluted culture plus any chemical supplements (IPBC, 0.5 μg/ml; NMCCC compounds, 133 μM) were transferred to 96-well plates. OD_600_ was measured at 20 h using a BioTek EL800 microplate spectrophotometer and effect strength calculated as above.

### Growth inhibition and cell toxicity assays for selected combinations of agents

*Growth inhibition assays in PDB broth:* the assays were performed as above with OD_600_ measured at 40 h. Combinatorial effect strength was calculated as follows: (% inhibition with IPBC + PL compound) minus (% inhibition with IPBC alone + % inhibition with PL compound alone). Combinations of interest were considered those with a combinatorial effect strength > 20.

*Growth inhibition assays on coated coverslips:* round cover glasses (13 mm diameter, thickness No. 1; VWR, Radnor, PA) were dip coated in 0.25% (w/v) sodium alginate, supplemented with inhibitors where indicated, and air-dried for an hour. A suspension of *T. virens* spores (10^6^ spores ml^−1^ from 7-day old PDA plates, suspended in nutrient salts solution [0.5% peptone (Oxoid), 0.3% yeast extract (Oxoid), 0.5% sodium chloride (Sigma), pH 6.8]) was sprayed onto the coated cover glasses and these were then placed spore-side upwards onto water agar [sterile distilled water, 2% (w/v) agar] in 12-well plates (Greiner, Kremsmünster, Österreich). Plates were incubated at 30 °C for 14 days before microscopic images were captured to assess growth of *T. virens* on the coated cover glasses.

*Cell toxicity assays:* human cells (TE671, rhabdomyosarcoma RD cell line) were cultured and toxicity to the cells was assayed using the CCK-8 reagent (Sigma) as described previously (Moreno-Martinez et al. 2015).

## Results

### Development of new compound combinations with BPR-approved IPBC, for fungal inhibition

**Fig. 1:**
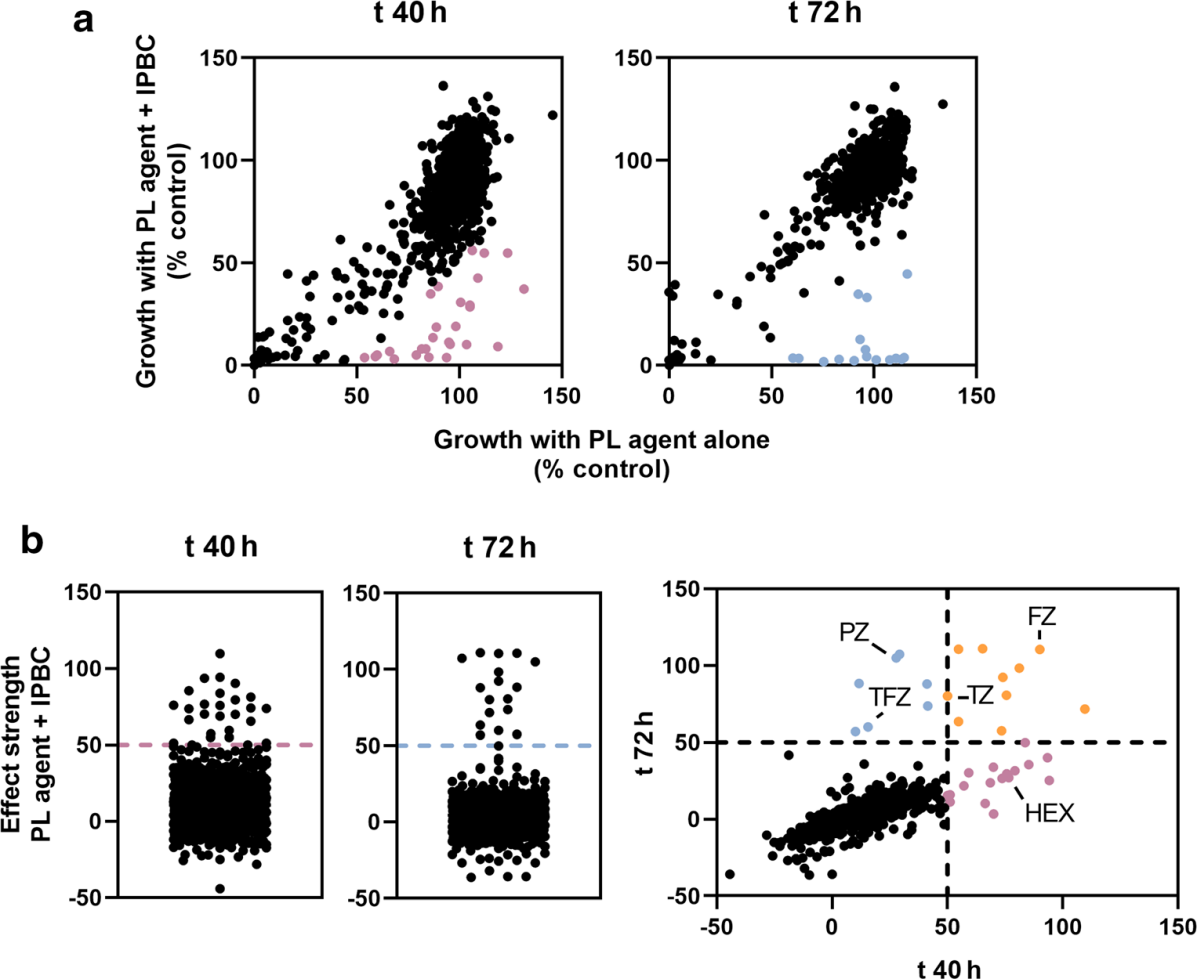
High-throughput chemical screening for agents that potentiate fungal growth inhibition by IPBC*.* (A) The scatterplots show the normalised growth yield (OD_600_) of *T. virens* for each PL compound in the absence (*x* axis) and presence (*y* axis) of IPBC, at 40 h and 72 h. The data are means from duplicate screens. (B) Effect strengths of the 1280 combinations; those that showed an effect strength >50 at 40 h are coloured in pink, at 72 h in blue (in A and B) and at both time points in orange (panel on the right ). Agents selected for further tests are indicated: TZ, thioridazine; FZ, flunarizine; TFZ, trifluoperazine; PZ, prochlorperazine; HEX, hexetidine. The full dataset is in Supplemental Table S1

The study was performed with the five fungi of the ASTM G21 group (*A. brasiliensis*, *A. pullulans*, *P. funiculosum*, *C. globosum*, *T. virens*), as these are the industry standard for determining resistance of synthetic polymeric materials to environmental fungi. This group was originally selected by the ASTM based on their affinities for using plasticizers, cellulose, lubricants, stabilisers, and colorants as growth substrates. A screen was performed against *T. virens*, with the Prestwick chemical library (PL), comprising 1280 FDA-approved compounds, in the absence and presence of a low concentration of the BPR-approved agent IPBC (i.e., 200 ng/ml, which produces less than 10% reduction of growth yield). *T. virens* was used for these screens as it gave the best reproducibility in growth assays and hence confidence in identifying the screen hits. A relatively low spore density (15,000 spores ml^−1^) was used to maximise sensitivity of detection of milder drug effects. Library compounds were supplied at 100 μM, the maximum concentration attainable while ensuring that the added volumes of the stock solutions (in dimethyl sulfoxide, DMSO) did not exceed 2% of the final volumes. Measurements of growth inhibition by the PL agents alone or in combination with IPBC were taken at two different time points, 40 h and 72 h, which correspond to early and later stationary growth phases and were shown previously to be suitable for detecting strong combinatorial effects (O’Brien et al. 2019). At 100 μM, ~90% of PL agents had minimal growth effects when supplied alone (i.e., less than 20% reduction of growth yield) (Fig. 1A, Supplemental Table S1). Forty-nine PL compounds gave more than 75% reduction of the *T. virens* growth yield after 72 h; these included 26 known fungicides including 16 azoles, and 3 antiseptics (Supplemental Table S1). When IPBC was included, effect strengths were calculated in order to identify combinations of potential interest, selected here as those that showed an effect strength >50 (i.e., where the difference between % growth with PL compound alone and the combination was >50). At 40 h, 27 combinations exhibited an effect strength >50, but only 10 of these retained this effect at 72 h (Fig. 1B, Table 1, Supplemental Table S1). At the later time point, a total of 17 combinations showed an effect strength >50 (Fig. 1B, Table 1, Supplemental Table S1). Of these, the seven that did not appear as potential hit combinations at 40 h included PL compounds which were effective on their own at that time point with a reduction of growth >55%. The most promising library compounds were selected for further tests based on three criteria: efficacy of *T. virens* growth inhibition in combination with IPBC at t 72 h; known modes of action (e.g., where several compounds had similar, known modes of action, certain examples were selected; Table 1); low relative cost (< £50 per gram; Sigma; Table 1). These were thioridazine hydrochloride (TZ), flunarizine dihydrochloride (FZ), trifluoperazine dihydrochloride (TFZ), and prochlorperazine dimaleate (PZ). The known antifungal hexetidine (HEX), which showed a promising effect in combination with IPBC at t 40 h, was also selected as hexetidine additionally has biocidal approval (Table 1).

**Table 1:** Hits from high-throughput chemical screening for agents that potentiate *T. virens* growth inhibition by IPBC at t 72 h.

	Effect strength^a^		Target^b^	Cost in £/g (Sigma reference)	Selected for further experiments^c^
	t 40 h	t 72 h			
Meclozine dihydrochloride	**65.2**	**111.0**	Histaminergic H1 receptor	813 (M0220000)	No *Expense*
Perphenazine	**54.8**	**110.5**	Dopaminergic receptors	30 (P6402-5G)	No *Similar target as PZ, TZ, and TFZ*
Flunarizine dihydrochloride	**90.1**	**110.4**	Ca2+ channel	34 (F8257-10G)	**Yes**
Sertindole	29.2	**107.2**	Dopaminergic receptors	7400 (ATE9594438278-250MG)	No *Expense;* *Similar target as PZ, TZ, and TFZ*
Prochlorperazine dimaleate	27.8	**105.0**	Dopaminergic receptors	10 (P9178-5G)	**Yes**
Demecarium bromide	**81.2**	**98.3**	Not listed	1192 (1169001-250MG)	No *Expense*
Astemizole	**74.0**	**92.2**	Histaminergic H1 receptor	5620 (A2861-50MG)	No *Expense*
Halofantrine hydrochloride	11.8	**88.2**	Not listed	9460 (H9414-50MG)	**(Yes)**
Fluphenazine dihydrochloride	41.1	**88.0**	Dopaminergic receptors	41 (F4765-5G)	No *Similar target as PZ, TZ, and TFZ*
Propidium iodide	**75.6**	**80.6**	Membrane	931 (P4170-1G)	No *Expense*
Thioridazine hydrochloride	**50.0**	**80.0**	Dopaminergic receptors	9 (T9025-25G)	**Yes**
Methiothepin maleate	41.5	**73.6**	Serotoninergic 5-HT receptors	Not available	No *Expense*
Daunorubicin hydrochloride	**109.6**	**71.5**	DNA	14,480 (30450-25MG)	No *Expense*
Suloctidil	**54.8**	**63.5**	Not listed	Not available	No *Expense*
Trifluoperazine dihydrochloride	15.6	**59.9**	Dopaminergic receptors	12 (T8516-10G)	**Yes**
Doxorubicin hydrochloride	**73.5**	**57.5**	DNA	19,400 (D1515-10MG)	No *Expense*
Zuclopenthixol dihydrochloride	10.2	**56.8**	Adrenergic alpha receptor	469,231 (Y0000534)	No *Expense*
Hexetidine	**76.6**	27.2	Membrane	12 (259187-10G)	**Yes**

^a^Effect strength ≥50 shown in bold

^b^Information from Prestwick Library Data Sheet

^c^Four compounds from the screen hits at 72 h were selected for further experiments based on low relative cost (< £50 per gram) and target (e.g., 6 compounds in the table are known to target dopaminergic receptors so only 3 of these were selected as representative for that class of inhibitors). As the approved biocide hexetidine showed potential at t 40 h, the agent was also selected. Halofantrine was selected for an experiment described in Fig. 4

### Spectrum of activity against environmental fungi of concern to the biocides industry

**Fig. 2:**
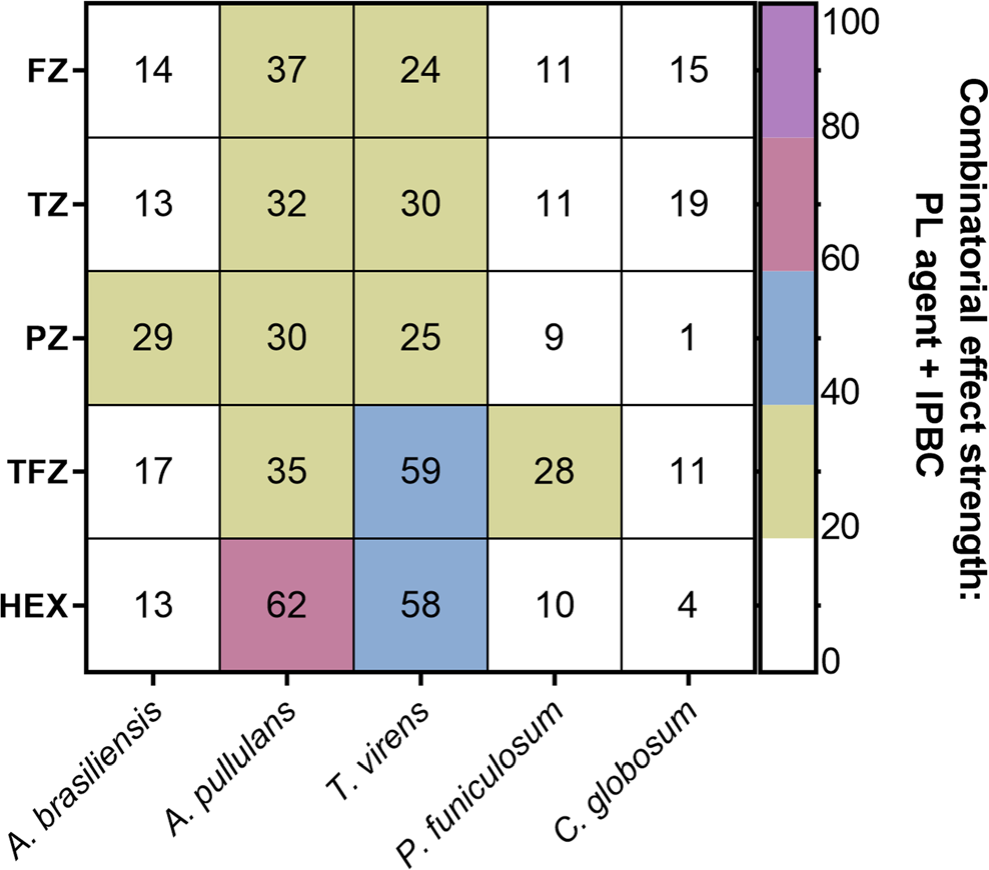
Library compound chemical interactions with IPBC in several environmental fungi. The colour gradient relates to the combinatorial effect strength for each combination, determined after 40 h growth of the fungus indicated. Combinatorial effect strength was calculated as described in the “Methods”. Data are means from at least three independent experiments. TZ, thioridazine, FZ, flunarizine, TFZ, trifluoperazine, PZ, prochlorperazine, HEX, hexetidine

To assess the spectrum of activity of the most promising combinations selected from the screen (Table 1), each of these was tested against five environmental fungi from the ASTM G21 group described above. Agents were supplied at sub-inhibitory concentrations (determined in preliminary assays) alone or in combination and growth was measured at 40 h. As agents in some instances showed an effect on their own, here the combinatorial effect strength was calculated slightly differently than effect strength above, i.e., (% inhibition with IPBC + PL agent) minus (% inhibition with IPBC alone + % inhibition with PL agent alone). The combinatorial effect strength of TFZ with IPBC was >25 against three environmental fungi (*A. pullulans*, *T. virens*, and *P. funiculosum*) (Fig. 2). HEX + IPBC showed a strong (>50) combinatorial effect against *A. pullulans* and *T. virens* (Fig. 2). Although other test fungi like *A. brasiliensis* did not score such an effect with this combination, it should be noted that *A. brasiliensis* is much more sensitive to either of these agents alone. Complete inhibition of *A. brasiliensis* growth occurred at concentrations (MICs 250 ng ml^−1^ and 250 μM for IPBC and HEX, respectively; not shown) that corresponded approximately to the sub-inhibitory concentrations used for *T. virens* (200 ng ml^−1^ and 200 μM, respectively). Therefore, application of combination dosages needed to control growth of the more resistant fungi (e.g., *T. virens*) could also inhibit more sensitive fungi (e.g., *A. brasiliensis*) regardless of a combinatorial effect.

**Fig. 3:**
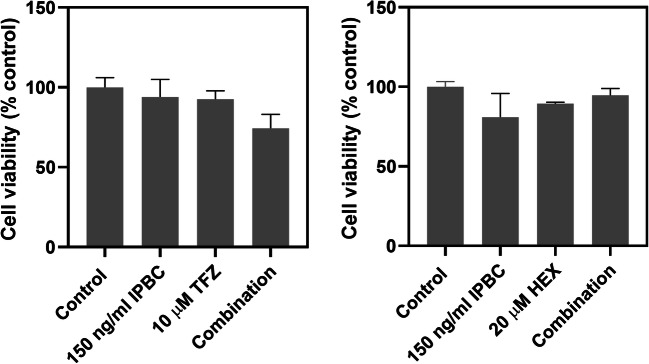
Trifluoperazine and hexetidine do not potentiate the effect of IPBC in a human cell line. Cells were incubated for 24 h in the presence of the compounds alone or in combination. Cell viability was then measured using a CCK-8 kit. The values are means ± SEM from three replicate experiments. The combinatorial effect strength for both combinations is <12. There were no significant differences between the conditions according to multiple comparisons (Tukey’s test) by one-way ANOVA

To assess specificity of the combinations for the fungi, the effects of the most promising combinations IPBC + TFZ and IPBC + HEX on human cell viability were tested. TFZ and HEX did not significantly potentiate the effect of IPBC against the human cell line, suggesting that the combinatorial effects were specific to the fungi (Fig. 3).

**Fig. 4:**
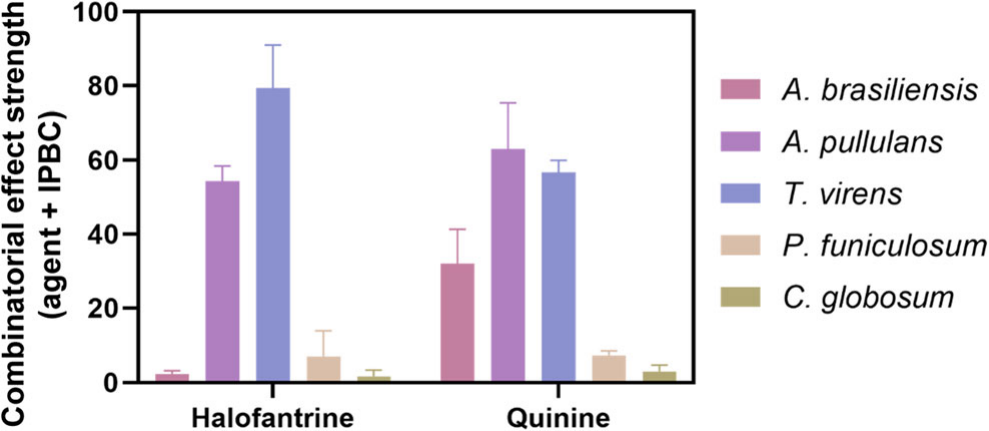
Halofantrine and quinine interactions with IPBC against the ASTM G21 fungi. After 40 h growth of the five indicated fungi, combinatorial effect strength was calculated as described in the “Methods” for the combinations halofantrine + IPBC and quinine + IPBC. The values are means ± SEM from at least three independent experiments

Ten of the 17 compounds highlighted earlier were relatively expensive and therefore might not be attractive as biocides (Table 1). These included halofantrine; halofantrine + IPBC had an effect strength of 88. Halofantrine is related to the cheap antimalarial drug quinine. Therefore, both antimalarials were tested in combination with IPBC, against the five ASTM G21 fungi (Fig. 4). Quinine potentiated the effect of IPBC against three of these environmental fungi while halofantrine potentiated IPBC action against two of them (*A. pullulans* and *T. virens*).

### Activity against *T. virens* within alginate material

**Fig. 5:**
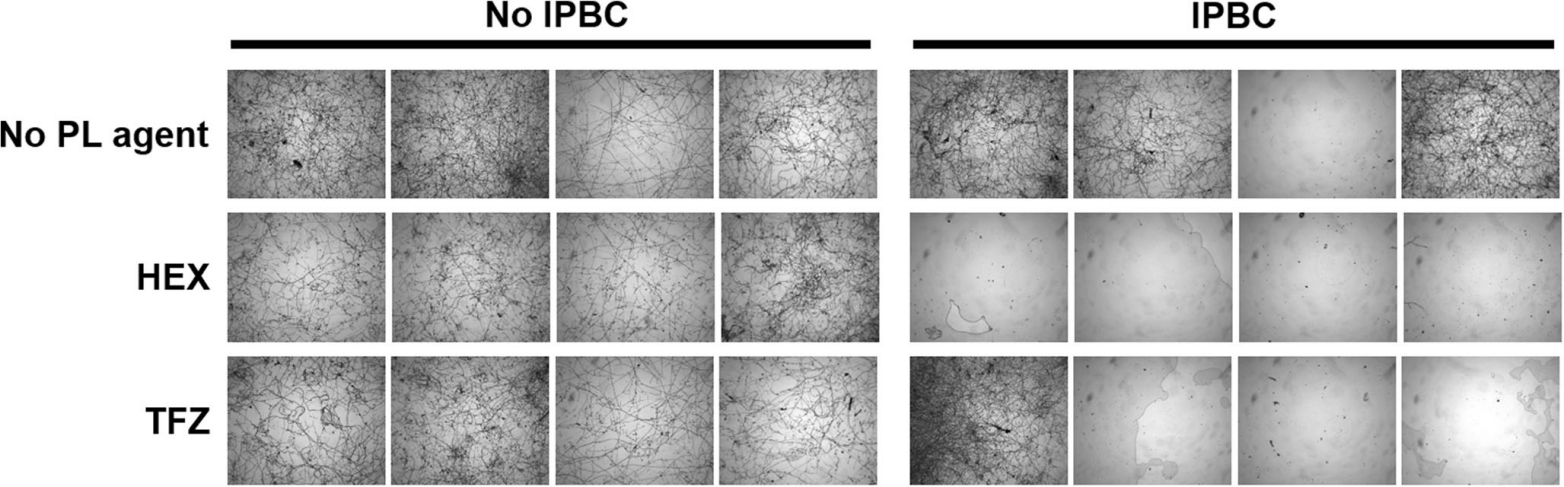
The activity of the combinations IPBC + HEX/TFZ is retained after coating on glass with sodium alginate. Round glass covers were coated with 0.25% (w/v) sodium alginate supplemented with 250 μg ml^−1^ IPBC, 15 μM HEX, and/or 15 μM TFZ alone or in combination. Spores of *T. virens* were then sprayed on to surfaces of the coated covers. Images were taken after 14 days. The four replicate images for each condition are from independent experiments

For potential applications, ideally biocide combinations would retain their efficacy in or on products to which they could be incorporated, e.g., polymers, thermoplastics, coatings, and construction materials. Here, as a proof of principle, the most promising fungicidal combinations, IPBC + TFZ and IPBC + HEX (IPBC + quinine was not assayed as quinine is not a PL compound), were incorporated in a biodegradable alginate polymer matrix that has shown potential as an antifungal and fungicide delivery system (Singh et al. 2009a; Singh et al. 2009b; Spadari et al. 2017). Agents were incorporated in 0.25% sodium alginate alone or in combination; alginate on its own did not exert fungal growth inhibition (Supplemental Fig. S1). Mixtures were used to dip-coat cover glasses before assessing effects on *T. virens* growth. Both the combinations IPBC + TFZ and IPBC + HEX retained strong inhibition of *T. virens* growth after incorporation within the alginate gel, when compared to the effects of the single agents alone (Fig. 5). There was some variation between certain independent experiments, but there was no sign of significant growth in 7 of the 8 experiments where the agents were supplied as combinations. As the experiments aimed to establish whether combination effects of the two agents were retained when in the alginate gel, and not to optimise the matrix, drug release properties from the gel were not evaluated. However, the data show that properties of combinatorial fungal inhibition were retained in the dip-coated alginate matrix, indicating that both drugs were available in sufficient concentrations over the time periods of the experiments to prevent fungal growth.

## Discussion

This study’s premise was that combination of the BPR-approved compound IPBC, with different FDA-approved agents may facilitate development of novel biocide combinations for combatting environmental fungi. These are the fungi that colonise commercial products and materials, causing their contamination and potential deterioration. Supporting the study’s hypothesis, several novel combinations were identified where antifungal efficacies of the individual agents were strongly potentiated. As biocides are typically used on or in products, their efficacy should be resilient in these applications, e.g., after incorporation to a polymer material. As proof of principle, an alginate polymer matrix was used to assess that potential for the combinations identified in this work. Alginate is a natural polymer that has been explored in antifungal and fungicide delivery systems, including with the carbamate thiram, due to its low toxicity, biodegradability, and low cost (Singh et al. 2009a; Singh et al. 2009b; Spadari et al. 2017). Recently, polymeric materials that prevent fungal attachment and colonisation passively (i.e., without killing the organism), including by *A. brasiliensis*, have been discovered (Vallieres et al. 2020a). Incorporation of the combinations to those polymers could potentially offer added protection from harmful fungi.

A high-throughput chemical library screening strategy (with the Prestwick library) proved effective here for identifying agents that strongly inhibit fungal growth when combined with IPBC. Among potential combinations of interest (based on criteria like relative efficacy, low cost, and mode of action), subsequent focus was mainly on trifluoperazine + IPBC and hexetidine + IPBC. The combinations were effective against at least two of the five environmental fungi from the industry-standard ASTM G21 group; their effect was greater than expected from the individual effects. As the combinations of interest were not effective against all of the fungi, this implies that, in potential applications, selection of appropriate treatments would need to be tailored to particular identified fungi. Moreover, the combinatorial effect observed against the fungi was not observed against human cells suggesting a fungal specificity. Criteria such as relative efficacy and low cost were not exhaustive as the study was primarily concerned with proof of principle. Other criteria like environmental stability would need to be evaluated to progress new biocide combinations to final application. Mode of action was also considered. Six of the 17 agents with an effect strength >50 after 72 h with IPBC are known to target dopaminergic receptors in humans, which contributed to us ruling out several of these. Ten of the 17 were not considered attractive as biocides as they were relatively expensive; among these, the antimalarial halofantrine is related to the cheap drug quinine, which was found to potentiate the effect of IPBC against three of the environmental fungi. This highlights the added possibility from the present study of considering hits that may be non-ideal, for different reasons, as providing chemical signatures for seeking related alternatives that may not have the same drawbacks, thus greatly expanding the potential array of chemical hits arising from this approach. This also shows that the present, high-throughput PL screen was by no means exhaustive. Also, only FDA-approved compounds were considered. A key advantage is that information on toxicology and pharmacokinetics from preclinical and clinical trials is already available, lowering regulatory barriers, development time, and cost. Therefore, the re-purposing of such agents for alternative uses has emerged as one strategy to facilitate development of antifungal therapeutic treatments (Holbrook et al. 2017; Robbins et al. 2015; Roemer and Krysan 2014; Spitzer et al. 2011).

Novel biocide combinations could also be identified by screening other ranges of compounds with IPBC. Recently, copper, which has BPR approval for some applications, and aminoglycoside antibiotics have both been found to act synergistically with fungicide carbamates such as thiram and ziram (Vallieres et al. 2018). As IPBC is also a carbamate, this suggests further chemistries with which IPBC could potentially be combined effectively. Synergy between IPBC and epoxy-amine oligomers from terpenes was observed against *T. virens* growth (O’Brien et al. 2019). To corroborate the wider potential for detecting novel combinations of interest with IPBC, an additional screen has been performed with 1000 compounds encompassing broad chemical space from the University of Nottingham Managed Chemical Compound Collection (NMCCC; which comprises >100,000 compounds in total). Encouragingly this additional screen, using the yeast *S. cerevisiae*, revealed four further compounds showing a combinatorial effect with IPBC (Supplemental Fig. S2; Supplemental Table S1).

As mentioned above, six of the 17 combinatorial effects observed at 72 h fungal growth involved a dopaminergic drug. Chemogenomic screens using yeast revealed that strains deleted in aromatic amino acid biosynthetic genes were hyper-sensitive to dopaminergic compounds (Ericson et al. 2008). Given that aromatic amino acids are precursors to dopamine, they considered that the sensitivity might reflect a blockage of aromatic amino acid uptake in yeast, requiring yeast to activate the corresponding biosynthetic pathways. Dopaminergic drugs like trifluoperazine are also known to inhibit the essential calcium-binding protein calmodulin (Butts et al. 2013; Prozialeck and Weiss 1982) and to perturb membrane, vesicle trafficking, and lipid biosynthesis pathways (Spitzer et al. 2011). Trifluoperazine has a synergistic action with fluconazole against several fungal pathogens (Spitzer et al. 2011). Membrane disruption caused by this agent may perturb ergosterol metabolism and therefore potentially increase susceptibility to accumulation of ergosterol intermediates, disturb fluconazole export by drug efflux pumps, and/or impair import of exogenous ergosterol (Kuo et al. 2010). Fluconazole also synergistically inhibits *Candida albicans* when combined with the calcium channel blocker flunarizine (Liu et al. 2016). Those data suggest the possibility that the BPR-approved fungicide (benzothiazol-2-ylthio) methyl thiocyanate (TCMTB), which also prevents fungal growth by disrupting ergosterol biosynthesis, could prove more potent in combination with compounds such as trifluoperazine and flunarizine. Chemogenomics (Dias et al. 2010) and proteomics (Santos et al. 2009) studies performed with yeast indicated that the fungicide mancozeb may cause alterations in amino acid metabolism and interference with membrane organisation and embedded transport systems. Given that IPBC is a carbamate, like mancozeb, it could be hypothesised that IPBC and dopaminergic drugs such as trifluoperazine target common processes, e.g., amino acid metabolism or membrane organisation, potentially explaining certain of the combinatorial effects observed in this study.

This study highlights new combinations of BPR-/FDA-approved compounds that could strongly inhibit the growth of key environmental fungi, including after incorporation to an alginate matrix. In combination, the compounds can be deployed at lower concentrations than if used singly, without impairing overall efficacy, so reducing concerns over chemical residues and toxicity. As the compounds in each combination have regulatory approval (at least for certain applications), this lowers development risk and allows a more rapid response to help meet the high demand for new biocides. The study suggests an effective strategy for improving fungicidal efficacies in biocide applications, using novel compound combinations.

## Supplementary Information

ESM 1 (XLSX 282 kb)

ESM 2 (PDF 104 kb)

## Data Availability

All data generated or analysed during this study are included in this published article and its supplementary information files.
