# Supplementary material for: Potentiated inhibition of Trichoderma virens and other environmental fungi by new biocide combinations
Source: Appl Microbiol Biotechnol. 2021 Mar 18;105(7):2867–75. doi: 10.1007/s00253-021-11211-3 (PMC8007513; doi:10.1007/s00253-021-11211-3)
Supplement: Supplementary file 2 — (PDF 104 kb) [file 253_2021_11211_MOESM2_ESM.pdf]

SUPPLEMENTARY FIGURES

Journal: **Applied Microbiology and Biotechnology**

Article Title: **Potentiated inhibition of *Trichoderma virens* and other environmental fungi by new biocide combinations**

Authors: Cindy Vallières[1], Cameron Alexander[2], Simon V. Avery[1*]

[1]School of Life Sciences, University of Nottingham, University Park, Nottingham NG7 2RD, UK

[2]School of Pharmacy, University of Nottingham, University Park, Nottingham NG7 2RD, UK

*Corresponding author: Simon.Avery@nottingham.ac.uk Tel: +44 (0)115 9513315

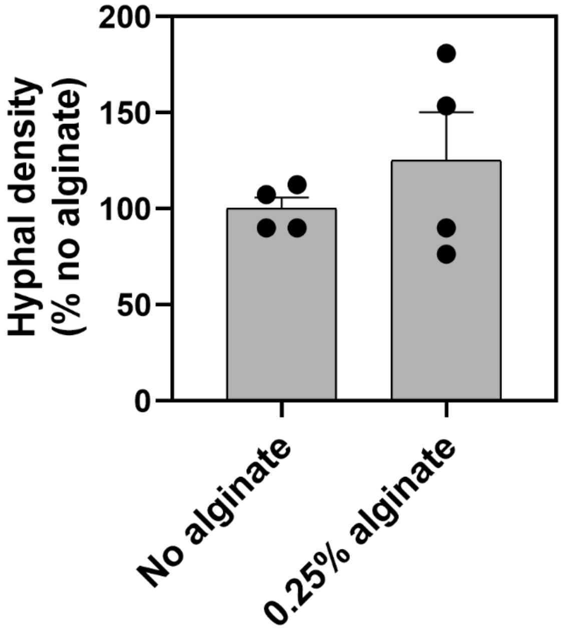

Fig. S1. Sodium alginate does not cause fungal growth inhibition. Hyphal density of *T. virens* on round glass covers coated with 0.25% (w/v) sodium alginate or not were analysed after 14 days using ImageJ. The values are means ± SEM from four replicate experiments. There was no significant difference according to Student's *t* test, two-tailed.

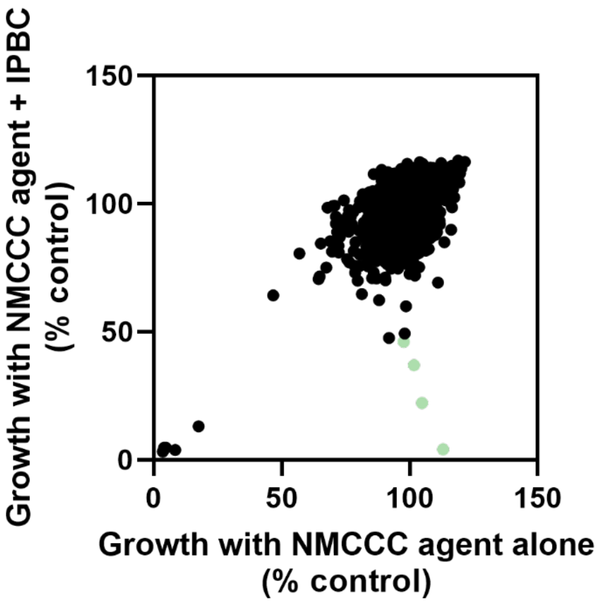

Fig. S2. High-throughput chemical screening data for NMCCC agents in combination with IPBC against yeast. *Saccharomyces cerevisiae* BY4743 was grown for 20 h in YPD broth in the absence or presence of the compounds, alone or in combination. As in Fig. 1, the scatterplots show the normalized growth of the yeast for each NMCCC compound in the absence (x axis) and presence of IPBC (y axis), after 20 h. The inset shows the effect strength of the 1,000 combinations; those that showed an effect strength >50 are coloured in green. The full dataset is in Table S1.
